# Astaxanthin Inhibits Matrix Metalloproteinase Expression by Suppressing PI3K/AKT/mTOR Activation in *Helicobacter pylori*-Infected Gastric Epithelial Cells

**DOI:** 10.3390/nu14163427

**Published:** 2022-08-20

**Authors:** Jimin Lee, Joo Weon Lim, Hyeyoung Kim

**Affiliations:** Department of Food and Nutrition, BK21 FOUR, College of Human Ecology, Yonsei University, Seoul 03722, Korea

**Keywords:** astaxanthin, gastric epithelial cells, *Helicobacter pylori*, metalloproteinase, mammalian target of rapamycin, phosphatidylinositol 3-kinase, serine/threonine protein kinase B

## Abstract

*Helicobacter pylori (H. pylori)* increases production of reactive oxygen species (ROS) and activates signaling pathways associated with gastric cell invasion, which are mediated by matrix metalloproteinases (MMPs). We previously demonstrated that *H. pylori* activated mitogen-activated protein kinase (MAPK) and increased expression of MMP-10 in gastric epithelial cells. MMPs degrade the extracellular matrix, enhancing tumor invasion and cancer progression. The signaling pathway of phosphatidylinositol 3-kinase (PI3K)/serine/threonine protein kinase B (AKT)/mammalian target of rapamycin (mTOR) is associated with MMP expression. ROS activates PIK3/AKT/mTOR signaling in cancer. Astaxanthin, a xanthophyll carotenoid, shows antioxidant activity by reducing ROS levels in gastric epithelial cells infected with *H. pylori*. This study aimed to determine whether astaxanthin inhibits MMP expression, cell invasion, and migration by reducing the PI3K/AKT/mTOR signaling in *H. pylori*-infected gastric epithelial AGS cells. *H. pylori* induced PIK3/AKT/mTOR and NF-κB activation, decreased IκBα, and induced MMP (MMP-7 and -10) expression, the invasive phenotype, and migration in AGS cells. Astaxanthin suppressed these *H. pylori*-induced alterations in AGS cells. Specific inhibitors of PI3K, AKT, and mTOR reversed the *H. pylori*-stimulated NF-κB activation and decreased IκBα levels in the cells. In conclusion, astaxanthin suppressed MMP expression, cell invasion, and migration via inhibition of PI3K/AKT/mTOR/NF-κB signaling in *H. pylori*-stimulated gastric epithelial AGS cells.

## 1. Introduction

*Helicobacter pylori* (*H. pylori*), a gram-negative and microaerophilic bacterium, selectively colonizes the mucous layer and surface of gastric tissues [1]. *H. pylori* upregulates oxidative stress, inflammation, invasion, and migration of gastric cells, triggering gastric carcinogenesis [2]. Among these factors, invasion is considered a critical feature in cancer development [3]. Previously, we showed that *H. pylori* induces the expression of matrix metalloproteinase (MMP)-10 in gastric epithelial cells and increases invasive cells [4]. These results demonstrate the possible role of MMPs in *H. pylori*-induced invasion of gastric epitheial cells.

MMPs are endopeptidases that can degrade the molecules of extracellular matrix (ECM) and non-matrix [5]. MMPs regulate tissue homeostasis and their activities are tightly regulated, from gene expression to pro-enzyme activation and interaction with tissue inhibitors of matrix metalloproteinases (TIMPs) [6]. Aberrant upregulation of MMPs breaks cell–cell and cell–ECM interactions, leading to cancer cell invasion and metastasis. Therefore, MMP expression could be a hallmark of cancer progression [7,8,9].

In gastric epithelial cells, *H. pylori* increased the expression of MMPs, such as MMP-1 [10,11,12], MMP-3 [13,14], MMP-7 [13,15,16], MMP-9 [17], and MMP-10 [11,18]. Among these, MMP-7 is the smallest enzyme of the MMP family and has many substrates, such as proteoglycans, laminin, and elastin. MMP7 induces activation of pro-MMP-2 and pro-MMP-9 for degradation of ECM [19,20]. Studies using antisense oligonucleotides of MMP-7 showed decreased motility in gastric glands that were isolated from *H. pylori*-infected patients [21]. Immunohistochemical analysis showed that MMP-7 was predominantly localized in cancer tissues, but not in noncancerous tissues of gastric mucosa [22,23]. MMP-10 also cleaves numerous ECM components and activates pro-MMP-1, pro-MMP-7, and pro-MMP-9 [24,25,26]. In cell cultures and human biopsies, *H. pylori* strains (cag A-positive) increase MMP-10 expression via epidermal growth factor receptor (EGFR)- and extracellular signal-regulated kinase (ERK)-mediated signaling pathways [18]. Recent study shows MMPs as specific biomarkers and treatment targets in *H. pylori*—associated stomach carcinoma invasion and metastasis [27]. In the patients and mice, *H. pylori* infection and interleukin-22 (IL-22) synergistically activated ERK and induced MMP-10 expression [28]. Therefore, MMP-7 and MMP-10 may be important MMPs in gastric cell invasion during *H. pylori*-associated gastric cancer development. 

The signaling pathway of phosphatidylinositol 3-kinase (PI3K)/serine/threonine protein kinase B (AKT)/mammalian target of rapamycin (mTOR) is a key regulator of cell growth and metabolism. Dysregulation of this pathway leads to cancer development, including invasion and metastasis [29,30]. PI3K/AKT/mTOR signaling is activated and induces MMP-9 expression in hepatocellular carcinoma [31] and MMP-2 expression in gastric cancer cells [32]. Expression of these MMPs mediates cancer cell invasion and migration [31,32]. Therefore, the inhibition of PI3K/AKT/mTOR signaling may be therapeutically important in treating gastric cancer.

MMP expression may be mediated by the activation of nuclear factor kappa-light-chain-enhancer of activated B cells (NF-κB) since the promoter region of MMP genes harbors a binding site for NF-κB. There is a crosstalk between the PI3K/AKT/mTOR signaling and NF-κB signaling pathway [33,34,35]. *H. pylori* increases oxidative stress, which affects signaling pathways such as NF-κB, activator protein-1, mitogen-activated protein kinases (MAPKs), and oxidative DNA damage-related signaling in gastric cells, leading to carcinogenesis [36,37]. *H. pylori* induces reactive oxygen species (ROS)-mediated activation of NF-κB for increasing cytokine level in gastric epithelial cells [38,39]. Moreover, ROS activates PIK3/AKT/mTOR signaling in the pathogenesis of cancer development [40]. Inhibition of PI3K/AKT activation and the maintenance of redox metabolism suppress cancer cell growth [41]. Therefore, we hypothesized that *H. pylori* stimulation might increase MMP-7 and MMP-10 expression through the PI3K/AKT/mTOR signaling and activation of NF-κB in gastric epithelial cells.

Astaxanthin is a red-orange, fat-soluble xanthophyll carotenoid that is abundant in aquatic organisms, including algae, krill, lobster, and salmon [42]. Structurally, it has an ionone ring at either end (polar region) and a series of carbon–carbon conjugated double bonds in the middle (nonpolar region). The ionone rings of the polar zone give this molecule the ability to quench singlet oxygen, while the polyene chain of the nonpolar zone creates more antioxidant dimensions to eliminate high-energy electrons from free radicals. This unique structure allows astaxanthin to exist in cell membrane—thereby acting both inside and outside the cell—and to possess outstanding antioxidant capacity [43,44]. Previously, we showed that astaxanthin activates peroxisome proliferator-activated receptor-γ (PPAR-γ) and induces the PPAR-γ target gene catalase, which reduces ROS in *H. pylori*–infected gastric epithelial cells [4]. β-carotene suppresses expression of MMP-10 by reducing ROS levels and inhibiting MAPKs activation in *H. pylori*-stimulated gastric epithelial cells [45]. A known antioxidant, N-acetyl-cysteine (NAC), suppressed expression of MMP such as MMP-9 and MMP-2, and cell invasion in pancreatic cancer cells [46], mammary epithelial cells [47], and gastric cancer cells [48]. Therefore, antioxidant nutrients may suppress *H. pylori*-induced MMP expression and cell invasion in gastric epithelial cells. Since astaxanthin has 10-fold stronger antioxidant activity than β-carotene (determined by in vitro fluorometric assay [49]), astaxanthin may exert a suppression effect on *H. pylori*-mediated MMP expression and cell invasion.

This study aimed to investigate whether (1) *H. pylori* stimulation upregulates both MMP-7 and MMP-10 expression, cell invasion, and migration via the PI3K/Akt/mTOR/NF-kB signaling pathways in gastric epithelial AGS cells and (2) astaxanthin has an inhibitory effect on *H. pylori*-mediated increase in MMP and invasive and migrated cells by suppressing PI3K/AKT/mTOR and NF-kB activation in gastric epithelial cells. To determine whether the PI3K/Akt/mTOR signaling pathway is an upstream signaling pathway for NF-kB activation, specific inhibitors of PI3K, AKT, and mTOR were treated to *H. pylori*-infected cells and NF-κB DNA-binding activity and MMP expression were assessed.

## 2. Materials and Methods

### 2.1. Reagents

Astaxanthin (Sigma-Aldrich, St. Louis, MO, USA) was dissolved in dimethyl sulfoxide (DMSO) and stored in nitrogen gas at −80 °C. Before treatment, the astaxanthin stock solution was diluted in fetal bovine serum (FBS). The PI3K inhibitor wortmannin (W1628, Sigma-Aldrich), AKT inhibitor SH-5 (ab141442, Abcam, Cambridge, UK), and mTOR inhibitor rapamycin (R8781, Sigma-Aldrich) were dissolved in DMSO.

### 2.2. Cell Culture and H. pylori Infection

The human gastric epithelial cell line AGS (gastric adenocarcinoma, ATCC CRL 1739) was purchased from the American Type Culture Collection (Rockville, MD, USA). The cells were grown in complete medium consisting of RPMI 1640 (Gibco, Grand Island, NY, USA) supplemented with 10% heat-inactivated fetal bovine serum (Gibco), 2 mM glutamine, 100 U/mL penicillin, and 100 µg/mL streptomycin (Sigma-Aldrich). The cells were cultured at 37 °C in a humidified atmosphere of 95% air and 5% CO_2_.

*H. pylori*, strain NCTC 11637 (*cag A*-positive, *vac A*-positive strain), was obtained from the American Type Culture Collection. The bacterium was inoculated onto chocolate agar plates (Becton Dickinson Microbiology Systems, Cockeysvile, MD, USA) at 37 °C under microaerophilic conditions using an anaerobic chamber (BBL Campy Pouch System, Becton Dickinson Microbiology Systems, Franklin Lakes, NJ, USA). AGS cells were cultured overnight to reach 80% confluency. Whole *H. pylori* was harvested from the chocolate agar plates, suspended in antibiotic-free RPMI 1640 medium supplemented with 10% fetal bovine serum, and then used to treat AGS cells. For the study on the effect of astaxanthin on MMP expression, AGS cells were treated with *H. pylori* NCTC 11637 at a bacteria:cells ratio of 50:1.

### 2.3. Experimental Protocol

To investigate the effect of astaxanthin on PI3K/AKT/mTOR signaling, MMP expression, and cell invasion, the cells (1.5 × 10^5^/2 mL, 7.0 × 10^5^/10 mL) were pre-treated with astaxanthin (at a final concentration of 1 or 10 µM) for 3 h and then infected with *H. pylori* for 8 h (for the analysis of *p*-PI3K, PI3K, *p*-AKT, AKT, *p*-mTOR, mTOR, and IκBα protein levels and NF-κB-DNA binding activity) or 24 h (for the analysis of MMPs mRNA and protein expression, Snail protein expression, and the invasive and migrated cell assay).

To assess the relation of PI3K, AKT, and mTOR signaling in NF-κB activation in *H. pylori*-infected cells, wortmannin (final concentration, 0.1 μM), SH-5 (final concentration, 1 μM), or rapamycin (final concentration, 0.1 μM) were pre-treated for 2 h before *H. pylori* infection. For each experiment, astaxanthin- or specific inhibitor-untreated cells received vehicle DMSO (0.01%) alone instead of astaxanthin or specific inhibitors.

Prior to the experiments, to determine the appropriate incubation time for MMP expression; activation of PI3K, AKT, and mTOR; and NF-κB, time-course experiments were performed after stimulating AGS cells with *H. pylori*. Briefly, the cells (1.5 × 10^5^/2 mL or 7.0 × 10^5^/10 mL) were infected with *H. pylori* at a ratio of 50:1 (bacteria:cells) for 6, 12, and 24 h to assess MMP expression; 2, 4, 6, and 8 h to assess PI3K/AKT/mTOR activation; or 4, 8, 12, 16 and 20 h to assess NF-κB activation and IκBα protein level.

In another set of experiments, *H. pylori* were treated to the cells at different infection ratio (bacteria:cells at 20:1, 0:1, or 100:1) for 24 h to assess the appropriate infection ratio of *H. pylori* for MMP expression.

In addition, epithelial cells that ectopically express Snail acquire invasive properties [50]. Snail controls epithelial–mesenchymal transitions (EMT) by repressing ECM protein E-cadherin by suppressing gene expression [50] or by binding to E-cadherin proximal promote [51].Therefore, Snail expression was determined in the cells treated with astaxanthin and stimulated with *H. pylori* to assess whether the inhibitory effect of astaxanthin on *H. pylori*-induced cell invasion is related to expression of Snail, an EMT marker.

### 2.4. Real-Time PCR Analysis

Real-time PCR analysis was followed by the method described previously [4]. Briefly, after total RNA isolation, it was reverse-transcribed into cDNA, which was amplified using primers for MMPs and β-actin. For the desired MMP-7 PCR products, the primers 5′-TCCCGCGTCATAGAAATAATG-3′ (forward primer) and 5′-AGGAATGTCCCATACCCAAAG-3′ (reverse primer) were used. For MMP-10, the primers 5′-CATTCCTTGTGCTGTTGTGTC-3′ (forward primer) and 5′-TGTCTAGCTTCCCGTTCACC-3′ (reverse primer) were used. For β-actin, 5′-ACCAACTGGGACGACATGGAG-3′ (forward primer) and 5′-GTGAGGATCTTCATGAGGTAGTC-3′ (reverse primer) were used. The synthesized cDNA was amplified, and β-actin served as a reference gene.

### 2.5. Invasion Assay

The invasive cell assay was performed by previously described method [4]. The number of invasive cells in the lower chamber, which migrated from the upper chamber separated with filter, was measured with a laser confocal microscope.

### 2.6. Wound-Healing Assay

Cells were seeded in 12-well plates and allowed to grow in complete medium until confluent. Then, individual wells were scratched with a 200-µL micropipette tip to create a denuded zone of constant width (1 mm). Complete media were removed from these cells and replaced with RPMI-1640 containing 4% FBS. The cells were pre-treated with astaxanthin (at a final concentration of 1 or 10 µM) for 3 h and then stimulated with *H. pylori.* To determine cell migration, cell images were captured at baseline (0 h) and after 24 h of *H. pylori* stimulation under phase-contrast microscopy. The final gap width of scratch was measured and calculated compared with the initial gap. Wound area was quantified with ImageJ software (NIH, Baltimore, MD, USA).

### 2.7. Measurement of Cell Viability

Cells in 24-well plates were pre-treated with astaxanthin (at a final concentration of 1 or 10 µM) for 3 h and then stimulated with *H. pylori* for 24 h. Viable cells were evaluated using 0.2% trypan blue exclusion (Sigma-Aldrich).

### 2.8. Western Blotting

The preparation of whole-cell extracts and western blot analysis were performed as previously described [4]. The proteins were determined using antibodies against MMP-7 (ab207299, Abcam, Cambridge, UK), MMP-10 (ab38930, Abcam), *p*-PI3K (#4228S, Cell Signaling Technology, Danvers, MA, USA), PI3K (#4292S, Cell Signaling Technology), *p*-AKT (#9271S, Cell Signaling Technology), AKT (#9272S, Cell Signaling Technology), *p*-mTOR (#5536S, Cell Signaling Technology), mTOR (#2972S, Cell Signaling Technology), IκBα (sc-371, Santa Cruz Biotechnology, Dallas, TX, USA), Snail (#3879, Cell Signaling Technology), and actin (sc-47778, Santa Cruz Biotechnology). After detection with the secondary antibodies, the proteins were determined using an enhanced chemiluminescence detection system. Actin served as a loading control. Densitometry data represent the mean ± standard error (SE) from three immunoblots and are shown as the relative density of protein bands normalized to the indicated protein (actin or total form of PI3K/AKT/mTOR).

### 2.9. Electrophoretic Mobility Shift Assay (EMSA)

NF-κB DNA-binding activity was determined using the gel shift oligonucleotide for NF-κB (5′-ACTTGAGGGGACTTTCCCAGGGC-3′; sc-2505, Santa Cruz Biotechnology, as previously described [4].

### 2.10. Immunofluorescence Staining

The cells were seeded on coverslips in 6-well plates, pre-treated with 10 µM astaxanthin for 3 h, and then stimulated with *H. pylori* for 8 h. Cells fixed in 4% formaldehyde were incubated with blocking buffer containing 1% BSA and 0.1% gelatin for 1 h, followed by anti-NF-κB p50 antibody (06-886, Upstate Biotechnology, Lake. Placid, NY, USA) for 1 h. After three washes with PBS, the cells were incubated with rhodamine-conjugated goat anti-rabbit IgG antibody (Santa Cruz Biotechnology) for 1 h. The cells were washed again, incubated with 5 μg/mL 4′,6-diamidino-2-phenylindole (DAPI) in blocking buffer for 30 min, and covered with Vectashield antifade medium. Images of cells stained with rhodamine (red) and DAPI (blue) were acquired using a laser scanning confocal microscope.

### 2.11. Statistical Analysis

All values are expressed as the mean ± SE of three independent experiments. For each experiment, four samples were placed in each group (total number of each group is 12). Analysis of variance (ANOVA), followed by Tukey’s post hoc test, was used for statistical analysis. Differences were considered statistically significant at *p*-values < 0.05.

## 3. Results

### 3.1. H. pylori Increases the Expression of MMP-7 and MMP-10 in AGS Cells

**Figure 1:**
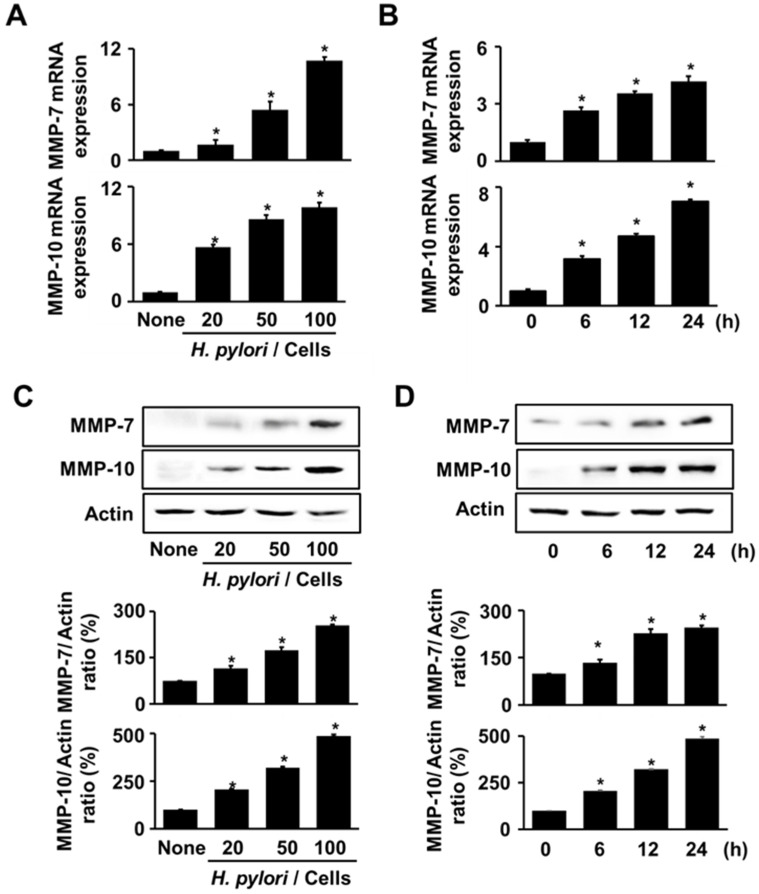
*H. pylori* increased MMPs expressions. (**A**,**C**) *H. pylori* was treated to the cells at the various ratios (*H. pylori*/cells) for 24 h. (**B**,**D**) *H. pylori* was treated to the cells at a 50:1 bacteria/cell ratio for the indicated durations. (**A**,**B**) mRNA expressions of MMP-7 and MMP-10 were analyzed via real-time PCR and normalized to β-actin mRNA expression. All values are expressed as the mean ± standard error (SE) of three independent experiments. (**C**,**D**) Protein levels of MMP-7 and MMP-10 were determined via western blot analysis. Actin was used as a loading control (upper panel). The densitometry data represent the mean ± standard error (SE) from three immunoblots and are shown as the relative density of protein bands normalized to actin (lower panel). * *p* < 0.05. vs. ‘None’ (uninfected cells) or 0 h.

To assess the appropriate infection ratio of *H. pylori* for MMP expression, AGS cells were stimulated with *H. pylori* at different infection ratios (bacteria:cells at 20:1, 50:1, or 100:1) for 24 h. Figure 1A,C show that *H. pylori* augmented the levels of MMP-7 and MMP-10 with an increase in the infection ratio. MMP expression at 50:1 and 100:1 bacteria/cell ratios was higher than that at 20:1. Even though the ratio of 100:1 showed the highest levels in MMP-7 and MMP-10 mRNA expression, the ratio of 50:1 was selected for time-course experiment. This is because MMP expression at 50:1 ratio was significantly increased. For the time-course experiments, the cells were stimulated with *H. pylori* for 6, 12, and 24 h. Both MMP-7 and MMP-10 increased with an increase in the incubation time expression at mRNA and protein expression (Figure 1B,D). Therefore, for the experiments on ASX effect for MMP expression, the cells were stimulated with *H. pylori* at an infection ratio (bacteria:cells) of 50:1 for 24 h.

### 3.2. H. pylori Activates PI3K, AKT, mTOR, and NF-kB in AGS Cells

**Figure 2:**
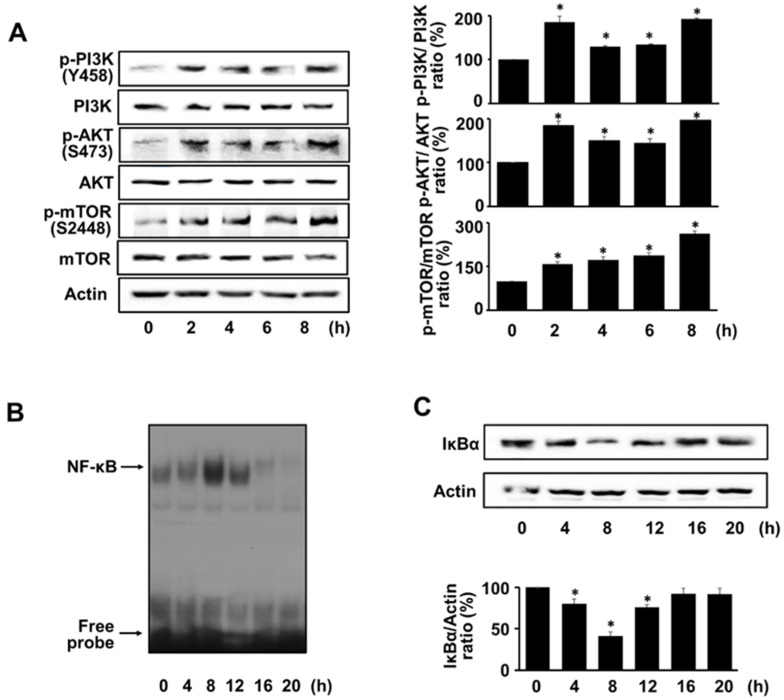
*H. pylori* activated PI3K, AKT, mTOR, and NF-kB. *H. pylori* was treated to the cells for the various durations. (**A**) PI3K, AKT, and mTOR levels (phosphor-specific and total forms) were determined using western blot analysis. Actin served as a loading control. (**B**) NF-κB DNA-binding activity was determined using electrophoretic mobility shift assay (EMSA). (**C**) Protein levels of IκBα were determined via western blot analysis. Actin served as a loading control (upper panel). The densitometry data represent the mean ± standard error (SE) from three immunoblots and are shown as the relative density of protein bands normalized to actin (lower panel). * *p* < 0.05 vs. 0 h.

To investigate whether *H. pylori* induces the PI3K/AKT/mTOR activation, the total and phosphor-specific forms of PI3K, AKT, and mTOR were detected using western blot analysis. *H. pylori* increased the phosphorylation of PI3K, AKT, and mTOR in AGS cells at 2 h and reached a maximum at 8 h. The total forms were not affected by *H. pylori* stimulation (Figure 2A). Whether *H. pylori* induced NF-κB activation or NF-κB DNA-binding activity and the expression level of the inhibitory protein IκBα were assessed via EMSA and western blot analysis, respectively. *H. pylori* increased NF-κB DNA-binding activity at 4, 8, and 12 h, and decreased it at 16 and 20 h (Figure 2B). The level of IκBα decreased at 4, 8, and 12 h and returned to the basal level at 16 and 20 h (Figure 2C). The increase in NF-κB DNA-binding activity and decrease in IκBα were maximal at 8 h. Therefore, to study the effect of ASX on the activation of PI3K, AKT, mTOR, and NF-kB, the cells were stimulated with *H. pylori* for 8 h.

### 3.3. Astaxanthin Suppresses Expression of MMP-7 and MMP-10 in H. pylori-Infected Cells

**Figure 3:**
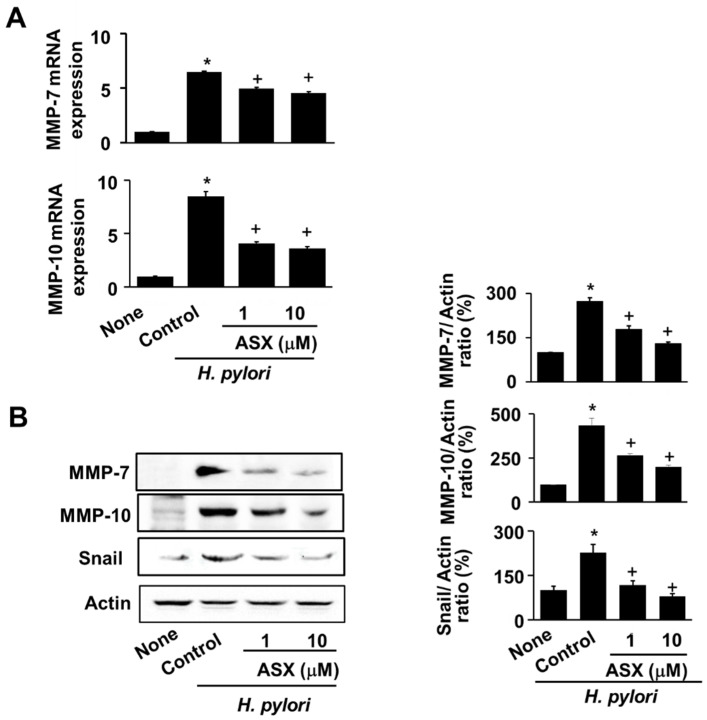
Astaxanthin inhibited MMPs and Snail expression in *H. pylori*-infected cells. Cells were pre-treated with the indicated concentrations of astaxanthin (ASX) for 3 h and infected with *H. pylori* for 24 h. (**A**) MMP-7 and MMP-10 mRNA expressions were analyzed using real-time PCR and normalized to β-actin mRNA expression. All values are expressed as the mean ± standard error (SE) of three independent experiments. * *p* < 0.05 vs. ‘None’ (uninfected cells without ASX treatment); + *p* < 0.05 vs. ‘Control’ (infected cells without ASX treatment). (**B**) The MMP and Snail protein levels were determined using western blot analysis, with actin as the loading control (left panel). The densitometry data represent the mean ± standard error (SE) from three immunoblots and are shown as the relative density of protein bands normalized to actin (right panel). * *p* < 0.05 vs. ‘None’ (uninfected cells without ASX treatment); + *p* < 0.05 vs. ‘Control’ (infected cells without ASX treatment). ASX, astaxanthin.

For MMPs expression, astaxanthin abrogated MMPs (MMP-7 and MMP-10) mRNA expression, which increased by *H. pylori* at 24 h (Figure 3A). Coinciding with the inhibitory effect of astaxanthin on MMP mRNA expression, astaxanthin reduced the protein levels of both MMP-7 and MMP-10 at 24 h (Figure 3B). Snail, a marker molecule of EMT, increased by *H. pylori* infection, which was inhibited by astaxanthin treatment in AGS cells at 24 h (Figure 3B).

### 3.4. Astaxanthin Suppresses the Activation of PI3K, AKT, mTOR, and NF-kB in H. pylori-Stimulated AGS Cells

**Figure 4:**
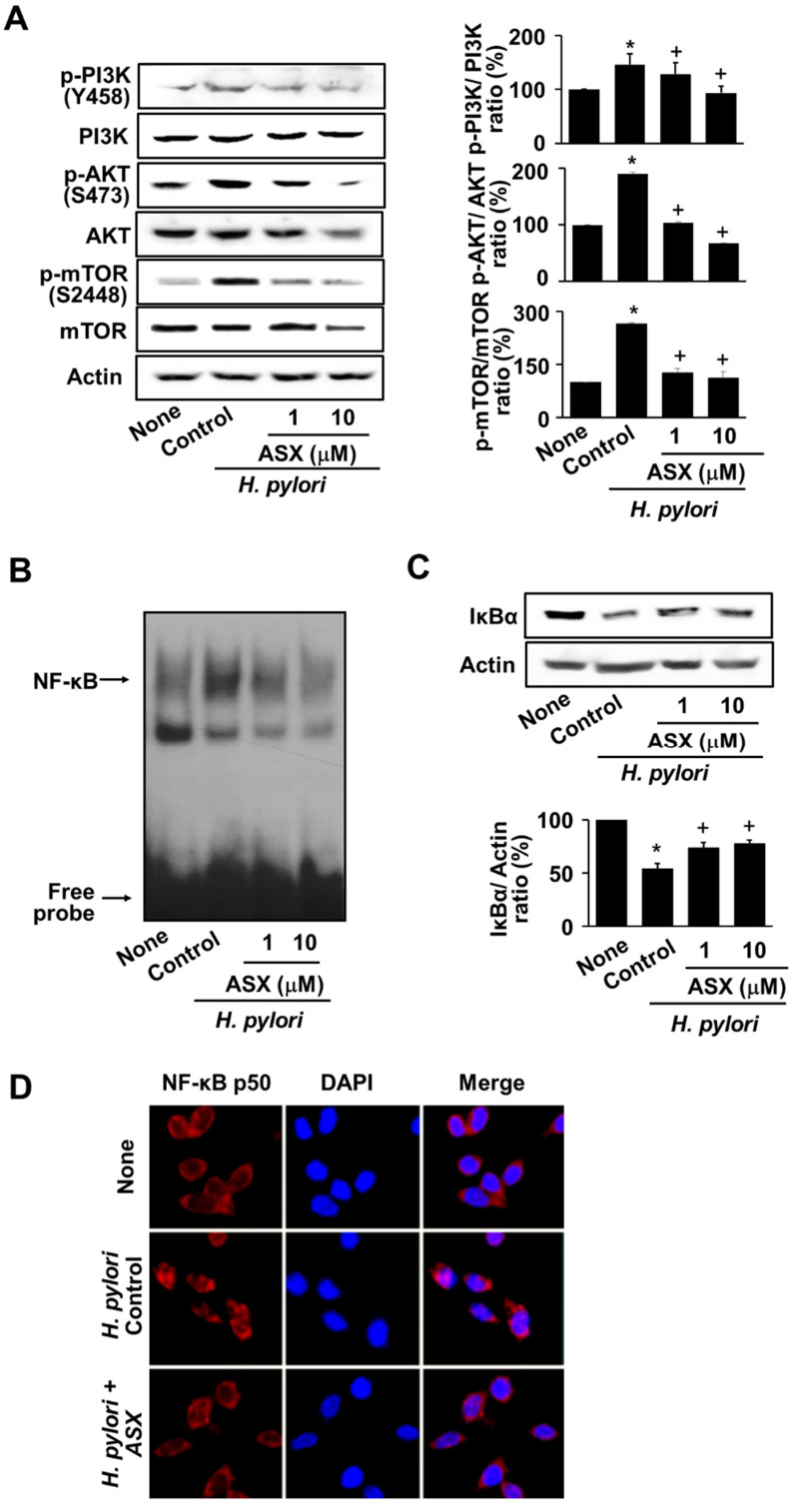
Astaxanthin suppressed activation of PI3K, AKT, mTOR, and NF-κB in *H. pylori*-infected cells. Cells were pretreated with the indicated concentrations of astaxanthin (ASX) for 3 h and infected with *H. pylori* for 8 h. (**A**) Phospho-specific and total forms of PI3K, AKT, and mTOR were examined using western blot analysis. Actin served as a loading control control (left panel). The densitometry data represent the mean ± standard error (SE) from three immunoblots and are shown as the relative density of *p*-PI3K, *p*-AKT, or *p*-mTOR protein bands normalized to total forms of PI3K, AKT, or mTOR (right panel). * *p* < 0.05 vs. ‘None’ (uninfected cells without ASX treatment); + *p* < 0.05 vs. ‘Control’ (infected cells without ASX treatment). (**B**) The DNA-binding activity of NF-κB was measured via electrophoretic mobility shift assay (EMSA). (**C**) IκBα levels were examined using western blot analysis. Actin served as a loading control (upper panel). The densitometry data represent the mean ± standard error (SE) from three immunoblots and are shown as the relative density of protein bands normalized to actin (lower panel). * *p* < 0.05 vs. ‘None’ (uninfected cells without ASX treatment); + *p* < 0.05 vs. ‘Control’ (infected cells without ASX treatment). (**D**) Cells and nuclei were stained with anti-NF-κB p50 antibody (red) and DAPI (blue), respectively. ASX, astaxanthin.

To examine whether the inhibitory effect of astaxanthin on MMP expression was mediated by the suppression of the PI3K/AKT/mTOR activation, PI3K/AKT/mTOR levels (phospho-specific form and total form) were determined. As shown in Figure 4A, astaxanthin markedly reduced the phospho-specific forms of PI3K, AKT, and mTOR in *H. pylori*-infected cells. We then examined whether astaxanthin suppressed the *H. pylori*–induced NF-κB activation. At 8 h, astaxanthin inhibited increased NF-κB DNA-binding activity and IκBα degradation in *H. pylori*-stimulated cells (Figure 4B,C). To confirm the inhibitory effect of astaxanthin on nuclear localization of NF-κB in *H. pylori*-infected AGS cells, nuclear localization of NF-κB was determined by immunofluorescence staining of NF-κB p50 (Figure 4D). *H. pylori* induced translocation of NF-κB p50 from cytosol to nucleus, which was inhibited by treatment of astaxanthin (10 µM) in AGS cells. Therefore, astaxanthin inhibits MMP-7 and MMP-10 expression by preventing the activation of PI3K/AKT/mTOR/NF-κB in *H. pylori*-stimulated cells.

### 3.5. Astaxanthin Inhibits H. pylori-Induced Invasion in AGS Cells

**Figure 5:**
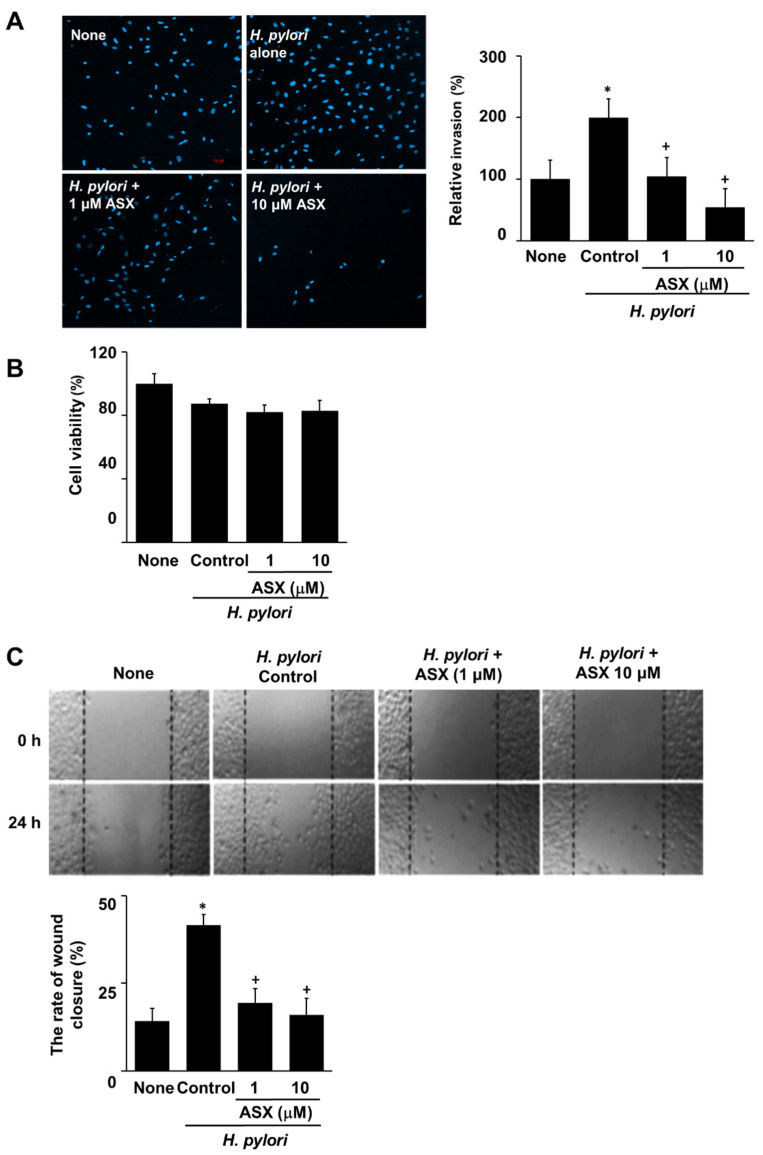
Astaxanthin inhibited cell invasion and migration in *H. pylori*-infected cells. AGS cells were pre-treated with the indicated concentrations of astaxanthin (ASX) for 3 h and then stimulated with *H. pylori* for 24 h. (**A**) Invasive cells were detected by staining them with DAPI on Matrigel-coated filters and visualizing them under a confocal laser scanning microscope (left panel). The graph represents the relative percentage of invasive cells. All values are expressed as the mean ± SE of three independent experiments (right panel). For each experiment, four samples were placed in each group (total number of each group is 12). The percentage of invasive cells in ‘None’ (uninfected cells without ASX treatment) was set as 100%. (**B**) Viable cells were evaluated using 0.2% trypan blue exclusion. The viable cell numbers of ‘None’ (uninfected cells without ASX treatment) were set at 100%. (**C**) Wound-healing assay was performed to detect the migration of cells. Representative images of wounds in AGS cells were taken at baseline (0 h) and 24 h later (upper panel). The graph represents the relative percentage of wound closure rate. Results were obtained from three independent measurements (lower panel). Data are presented as the mean ± standard error of the mean. For each experiment, four samples were placed in each group (total number of each group is 12). * *p* < 0.05 vs. ‘None’ (uninfected cells without ASX treatment); + *p* < 0.05 vs. ‘Control’ (infected cells without ASX treatment). ASX, astaxanthin.

Finally, we determined the suppressive effect of astaxanthin on cell invasion in *H. pylori*-stimulated cells. Briefly, AGS cells were infected with *H. pylori* in the presence or absence of astaxanthin and the prevalence of the invasive phenotype was analyzed. *H. pylori* increased invasive cell numbers, that was suppressed by astaxanthin at 24 h (Figure 5A). To determine whether *H. pylori* stimulation and astaxanthin treatment affect cell viability at 24 h culture, viable cell numbers were measured. Neither *H. pylori* nor astaxanthin affect cell viability (Figure 5B). To measure whether *H. pylori* induces migration of AGS cells, wound-healing assay was conducted. The migration rate can be expressed as the percentage of wound closure. Images were captured from the initial point of cell migration at 0 h and at a scratch after 24 h. *H. pylori* increased the rate of wound closure percentage, which was dose-dependently suppressed by astaxanthin at 24 h (Figure 5C).

### 3.6. Specific Inhibitors for PI3K, AKT, and mTOR Reversed the H. pylori-Induced NF-κB Activation, IκBα Degradation, and MMPs Expression in AGS Cells

**Figure 6:**
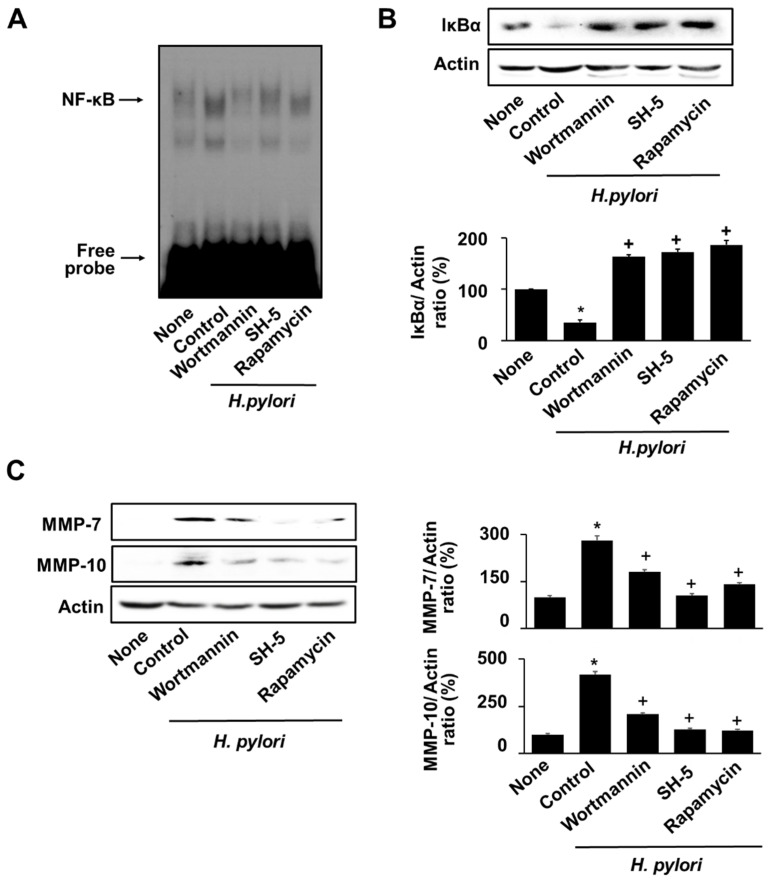
Inhibitors of PI3K, AKT, and mTOR suppressed NF-κB activation, IκBα degradation, and MMPs expression in *H. pylori*-infected cells. 0.1 µM wortmannin, 1 µM SH-5, or 0.1 µM rapamycin was pretreated to the cells. After 2 h, *H. pylori* was treated to the cells and cultured for 8 h. (**A**) DNA-binding activity of NF-κB was measured via EMSA. (**B**) Protein levels of IκBα were measured via western blot analysis. Actin was used as a loading control (upper panel). The densitometry data represent the mean ± standard error (SE) from three immunoblots and are shown as the relative density of protein bands normalized to actin (lower panel). (**C**) The protein levels of MMP-7 and MMP-10 were determined using western blot analysis. Actin was used as a loading control (left panel). The densitometry data represent the mean ± standard error (SE) from three immunoblots and are shown as the relative density of protein bands normalized to actin (right panel). * *p* < 0.05 vs. ‘None’ (uninfected cells without inhibitor treatment); + *p* < 0.05 vs. ‘Control’ (infected cells without inhibitor treatment).

To determine the association of PI3K, AKT, and mTOR in NF-κB activation and MMPs expression in *H. pylori*-infected cells, *H. pylori*-infected cells were pre-treated with wortmannin (PI3K inhibitor), Src-homolog 5 (SH-5, AKT inhibitor), and rapamycin (mTOR inhibitor) 2 h prior to *H. pylori* infection. These inhibitors suppressed the *H. pylori*–induced NF-κB activation and IκBα degradation (Figure 6A,B), suggesting that NF-κB activation is downstream signaling of PI3K/AKT/mTOR pathway in *H. pylori*-stimulated cells. As shown in Figure 6C, inhibitors of PI3K, AKT, and mTOR suppressed expression of MMP-7 and MMP-10 in *H. pylori*-infected cells.

## 4. Discussion

Oxidative stress caused by *H. pylori* infection induces hyperplasia and invasion of gastric epithelial cells, leading to pre-cancerous lesions [52]. Invasion and metastasis are hallmarks of cancer, and MMPs are known to induce these processes by degrading the ECM and cell–cell adhesion molecules. Overexpression of MMPs has been found in patients with *H. pylori*-related gastric cancer and in gastric epithelial cells stimulated with *H. pylori* [10,11,12,13,14,15,16,17,18]. Therefore, targeting MMPs could serve as a critical diagnostic factor in gastric carcinogenesis related to *H. pylori* infection.

ROS activates PIK3/AKT/mTOR signaling in the pathogenesis of cancer [40,53]. ROS activate the PI3K pathway by inhibiting the activity of its negative regulator phosphatase and tensin homolog deleted on chromosome 10 (PTEN) [54,55]. The inhibition of PI3K/AKT signaling and regulation of redox metabolism also inhibit cancer cell growth [56].

The present study was purposed to investigate whether *H. pylori*-induced MMP expression is mediated with the PI3K/AKT/mTOR signaling axis in gastric epithelial cells. Our findings show that *H. pylori* stimulation upregulated the expression of MMP-7 and MMP-10, cell invasion, and migration in AGS cells, indicating that *H. pylori* treatment affects the gene expression of MMPs, resulting in an invasive cell phenotype and migration. Furthermore, our results demonstrate that MMP expression is mediated with the activation of PI3K/AKT/mTOR in *H. pylori*-stimulated cells.

Upon activation through the PI3K/AKT/mTOR axis, the transcription factor NF-κB is separated from IκB through the activation of IKK, which phosphorylates IκB and translocates to the nucleus to participate in the expression of MMPs [33,34,35]. Here, we show that *H. pylori* stimulation induced IκB degradation and increased the DNA-binding activity of NF-κB. Thus, we demonstrated that *H. pylori* stimulation induced MMP expression via PI3K/AKT/mTOR-mediated NF-κB activation. In PTEN-inactive prostate cancer cells, the AKT-dependent activation of mTORC1 component Raptor is associated with IKKα [35]. The mTOR inhibitor rapamycin also inhibits IKK kinase activity, probably due to the dissociation of Raptor from the mTOR complex [56]. This study demonstrated that mTOR phosphorylates IκBα and activates NF-κB.

To determine the relation of PI3K/AKT/mTOR signaling and NF-κB activation, we used the specific inhibitors and determined the changes in *H. pylori*-induced NF-kB activation. Wortmannin, the most widely used PI3K inhibitor, was initially introduced for its anti-inflammatory capability [57]. Norman et al. [58] reported that it is an irreversible PI3K inhibitor that covalently attaches to lysine residues in the ATP-binding site of the p110 kinase domain. In AGS cells, wortmannin inhibited PI3K-mediated branching morphogenesis in response to gastrin-cholecystokinin B receptor stimulation [59]. Src-homolog 5 (SH-5) is a specific inhibitor of AKT that does not affect upstream kinases, such as PDK-1 [60]. SH-5 effectively blocks AKT activation and induce apoptosis in myeloid and lung adenocarcinoma and embryonic kidney cells [61]. Rapamycin inhibits mTOR by destabilizing the mTOR–Raptor complex, which is crucial for mTOR activation [62]. Rapamycin inhibited activation of mTOR in gastric cells stimulated with *H. pylori* [63]. In relation of the effects of wortmannin, SH-5, and rapamycyin, wortmannin inhibited phosphorylation of Akt and mTOR as well as PI3K [64,65]. A specific AKT inhibitor SH-5 inhibited mTOR activation [66] and AKT [60,61]. Rapamycin blocks phosphorylation of mTOR but did not affect phosphorylation of PI3K and AKT in several cell lines [67,68]. These findings show that mTOR is the downstream signaling of PI3K and AKT.

In the present study, PI3K and AKT activation was earlier than mTOR activation. This finding was supported by previous studies showing that a peak in mTOR phosphorylation appeared after several hours of a peak in Akt phosphorylation [69,70]. Regarding the mechanism of activation for PI3K/AKT/mTOR signaling, activated PI3K catalyzes the phosphorylation of PI(4,5)P2 to form PI(3,4,5)P3. It recruits Akt to the cell plasma membrane. Akt binds to PI(3,4,5)P3 phospholipid via its N-terminal PH domain, which is required for its recruitment to the cell plasma membrane. Upon membrane recruitment, Akt is phosphorylated by 3-phospho inositide-dependent protein kinase-1 (PDK1) at Thr308 in the activation loop of the kinase domain, in turn leading to Akt activation. Activated Akt indirectly activates mTOR by direct phosphorylation of the tumor suppressor TSC2 at S939 and T1462. TSC2 forms a heterodimeric complex with TSC1, and phosphorylation of TSC2 at these sites inhibits the GTPase-activating protein (GAP) activity of this complex. Because TSC2 suppresses the activity of the Ras-related GTPase Rheb, a selective activator of mTORC1, inhibition of TSC2 by Akt results in phosphorylation and activation of mTOR. Therefore, Akt phosphorylation by PI3K is followed by phosphorylation of mTOR.

Regarding the virulence factor of *H. pylori, vacA* (vacuolating cytotoxin gene) [71] and *cag* (the cytotoxin-associated gene) [72] are associated with peptic ulcer in Australia [73] and USA [71] and gastric dysplasia and cancer in USA [74] and Saudi Arabia [75]. In Korea, *cagA*-producing *H. pylori* are associated with increased risk of gastric cancer [76].

Peek et al. [77] reported another *H. pylori* gene, *iceA* (induced by contact with epithelium). There are two different alleles of this gene (*iceA1* and *iceA2*). The function of *iceA* is unknown. However, carriage of *iceA1* was shown to be weakly but significantly associated with peptic ulcer in studies of *H. pylori* strains from Netherlands [78], USA [77], and Japan [79].

Also, *cagA*, *vacA*, and *iceA1* were more frequent in follicular gastritis in a Colombian population at high risk for gastric cancer [80] and peptic ulcer in the Netherlands [78]. In contrast, Ito et al. [81] showed that *iceA* does not participate in the pathogenesis of peptic ulcer in Japan. Soltermann et al. [82] demonstrated that *cagA* and *vacA* are not related to the presence of lymphoid follicles. Pan et al. [83] showed that *vac A* is not associated with clinical gastric diseases such as gastritis and peptic ulcer. Therefore, different combinations of bacterial and/or human factors (host factors) may be critical determinants of disease.

In the present study, we found that *H. pylori* strain NCTC 11637, *cagA+*, *vac A+,* and *iceA+* strain increased MMP-7 and MMP-10 expression and invasive phenotype of gastric epithelial cells. These findings are supported by Lv et al. [28], which reported MMP-10 in *H. pylori* NCTC 11637 (*cagA+*) *H. pylori* but not in *cagA*-knockout mutant *H. pylori* NCTC 11637 (Δ*cagA*)–infected mice. Moreover, *cagA*-dependent *H. pylori* increased MMP-7 in HT29 colon cancer cells [15] and MMP-10 in gastric epithelial cells [16,18]. Jang et al. [10] showed that *cagA+* and *vac A+ H. pylori* increased MMP-10 in MGC-803 human gastric cancer cell line. These studies demonstrate that *cagA* and *vacA* may contribute to the expression of MMP-7 and MMP-10 in gastric epithelial cells. More studies are needed to determine the role of *iceA* on MMP expression in gastric epithelial cells.

Even though the combination of *H. pylori* genetic factors and other host factors may be important for gastric cancer development, *H. pylori* infection tends to increase the incidence of gastric cancer. Therefore, dietary supplements or dietary intake of antioxidant nutrients such as astaxanthin may be beneficial for preventing *H. pylori*-associated gastric cancer development.

Astaxanthin shows antioxidant and anticancer properties. In our previous studies, astaxanthin reduced ROS by activating PPAR-γ and inducing the PPAR-γ target gene catalase, which inhibited mitochondrial dysfunction and IL-8 expression in *H. pylori*-stimulated gastric epithelial cells [45]. Astaxanthin inhibits the phosphorylation of AKT and mTOR, which induces the activation of autophagy and inhibition of apoptosis in *H. pylori*-infected gastric epithelial cells [84]. ROS activate inflammatory signaling pathways such as MAPK and JAK/STAT and activate NF-kB and AP-1 to produce inflammatory mediators including cyclooxygenase-2, inducible nitric oxide synthase, IL-8, and monocyte chemoattractant protein-1 in *H. pylori*-stimulated gastric epithelial cells [39,85,86]. Therefore, antioxidant activity of astaxanthin may suppress ROS-mediated inflammatory signaling pathways and expression of the inflammatory mediators in gastric epithelial cells, resulting in suppression of gastric inflammation development. In addition, ROS induce gastric MMP expression, which activates invasion and metastasis, leading to gastric cancer progression. These studies suggest that astaxanthin may be useful for preventing gastric inflammation and gastric cancer via its antioxidant activity. Thus, astaxanthin can be used as a new pharmacological tool for preventing and treating *H. pylori*-associated gastric diseases including inflammation and cancer.

For the studies on astaxanthin and MMP expression and cell invasion, astaxanthin inhibited breast cancer cell migration compared to normal breast epithelial cells [87]. Furthermore, Chen et al. [88] demonstrated that astaxanthin reduced the expression of MMP-1, MMP-3, and MMP-13 through the inhibition of p38 and ERK1/2 phosphorylation as well as IκBα degradation in IL-1β-stimulated chondrocytes. Here, we found that astaxanthin successfully decreased MMP-7 and MMP-10 expression and the invasive cells by downregulating the PI3K/AKT/mTOR signaling and suppressing activation of NF-kB in *H. pylori*-infected cells.

The NF-κB family of transcription factors has an essential role in inflammation and innate immunity. NF-kB DNA-binding activity begins to decrease after stimulation. Several studies demonstrated that NF-kB DNA-binding activity reached a maximal induction within 2–6 h, and then decreased by 12–16 h [89,90,91]. In the present study, *H. pylori* increased NF-kB DNA-binding activity at 4–16 h and decreased after 16h in AGS cells.

In the present study, Snail expression increased from *H. pylori* infection. This finding is supported by previous studies [92,93]. Ngo et al. [92] showed that *H. pylori* induces expression of Snail through ROS-mediated activation of Erk in gastric epithelial AGS cells. Lee et al. [93] found that *H. pylori cagA* promotes Snail-mediated EMT and decreases E-cadherin, resulting in increased migration of AGS cells. Since astaxanthin inhibited Snail expression, the inhibitory effect of astaxanthin on cell migration may be related to increased Snail expression in *H. pylori*-stimulated AGS cells.

The limitation of the present study is that only one cell line of AGS cells was used to determine the inhibitory effect of astaxanthin on MMP expression, cell invasion, and migration and its signaling of PI3K/AKT/mTOR/NF-kB axis in *H. pylori*-stimulated cells. Further study should be performed using more gastric epithelial cell lines to prove the present findings.

## 5. Conclusions

Astaxanthin suppresses MMP expression, cell invasion, and migration via suppression of the PI3K/AKT/mTOR signaling pathway and NF-κB activation in *H. pylori*-stimulated cells. Therefore, dietary supplementation with astaxanthin may be beneficial for preventing *H. pylori*-induced gastric cancer cell invasion.

## Data Availability

All data generated or analyzed during this study are included in this published article.
